# F210. EXAMINING PATTERNS AND PREDICTORS OF INDIVIDUAL RESPONSE TO COGNITIVE REMEDIATION THERAPY

**DOI:** 10.1093/schbul/sby017.741

**Published:** 2018-04-01

**Authors:** Maree Reser, Susan Rossell

**Affiliations:** Swinburne University of Technology

## Abstract

**Background:**

People with serious mental health problems, particularly those with a diagnosis of schizophrenia and related disorders, often report difficulties in concentration, attention and memory. These cognitive problems can make day-to-day functioning more difficult, and are one of the strongest predictors of future persisting disability. Cognitive remediation therapy (CRT) has been shown to be moderately effective in helping people to strengthen such cognitive skills as processing speed, attention, working memory and auditory and visual memory. However, the same evidence also suggests that there is significant variability in response, with around 40–60% of CRT participants not benefiting. Traditional group analysis often masks such variability. To maximise the effectiveness of CRT, it is important to identify individual patterns of cognitive response and factors that predict such patterns.

**Methods:**

Twenty-two community-based individuals (12 male) with a mean age of 38.14 years (SD 9.85), diagnosed with schizophrenia (n = 15), schizoaffective disorder (n = 6) or schizophreniform (n = 1), completed a minimum 24-session cognitive remediation intervention in a single arm trial using Posit Science’s visual intensive program. Measures of premorbid and current IQ were administered at baseline and blood or saliva to perform genetic analysis was collected. The MCCB was administered pre- and post-intervention to evaluate cognitive response to CRT. To determine individual change at a MCCB cognitive domain and composite level, reliable change indices were calculated at the 95% confidence interval, adjusted for practice effects. Individuals were categorised according to whether there was a) no evidence of change, b) reliable change in at least one domain with maintenance across other domains, or c) evidence of a decline in cognitive functioning or a mix of decline and improvement. Correlates of group membership and individual patterns of response were examined.

**Results:**

Of the 22 participants, 11 experienced reliable change in at least one cognitive domain, 8 participants experienced no change, and 3 participants experienced either a decline (n = 1) or a mix of improvement and decline across distinct cognitive domains. Improvements were seen in attention/vigilance (18%, n = 4), processing speed (14%, n = 3), cognitive composite (14%, n = 3), reasoning and problem solving (9%, n = 2), working memory (9%, n = 2), verbal learning (5%, n = 1), visual learning (5%, n = 1), and social cognition (5%, n = 1). For a majority of improvers (n = 10), change was limited to a single domain.

**Discussion:**

Cognitive remediation has the potential to strengthen cognitive skills that underpin improvements in functioning. In line with other studies, using a stringent measure of reliable change, 50% of study participants showed improvement in at least one cognitive domain. Given the small sample size further analysis will examine select cognitive, genetic and learning potential correlates (variables selected for this analysis to be drawn from a recent systematic review from this team) with individual patterns of cognitive response to CRT.

